# Silicon isotopic fractionation under water stress in *Sorghum bicolor*: evidence from in situ morphotype-specific phytolith analysis

**DOI:** 10.1007/s11104-025-07633-x

**Published:** 2025-06-27

**Authors:** Francesca D’Agostini, Daniel A. Frick, Alessandra Varalli, Abel Ruiz-Giralt, Marco Madella, Carla Lancelotti

**Affiliations:** 1 CASEs Research Group, Department of Humanities, University Pompeu Fabra, 08005 Barcelona, Spain; 2 DIADE Unit, University of Montpellier, IRD, 34394 Montpellier, France; 3 Present Address: Kew Royal Botanic Gardens, London, TW9 3DS UK; 4 GFZ German Research Centre for Geosciences, Section 3.3 Earth Surface Geochemistry, 14473 Potsdam, Germany; 5 Present Address: Paleoceanography and Marine Geology, Institute of Geosciences, Kiel University, 24118 Kiel, Germany; 6 Present Address: Aix Marseille Univ, CNRS, Minist Culture, LAMPEA, 13094 Aix-en-Provence, France; 7 ICREA, 08010 Barcelona, Spain; 8 School of Geography, Archaeology and Environmental Studies, University of Witwatersrand, Johannesburg, 2000 South Africa

**Keywords:** Phytoliths, Si isotope, FsLA-MC-ICP-MS, Sorghum, Water stress

## Abstract

**Background and Aims:**

Phytolith studies are still facing numerous challenges regarding the available notions of Si absorption from soil and its deposition in aerial organs. This study shows how plant water availability affects the biosilica content and silicon isotopic composition of phytoliths in sorghum (*Sorghum bicolor*).

**Methods:**

Phytoliths were extracted from different plant parts of crops grown experimentally in lysimeters under water stress (WS) and well-watered (WW) conditions and analysed for silicon isotopic composition using femtosecond laser ablation multi-collector inductively coupled plasma-mass spectrometry (fsLA-MC-ICP-MS). This method provided precise isotope ratios of individual phytolith morphotypes.

**Results:**

Results indicate that while Si isotopic composition largely reflects watering conditions, single morphotypes present major differences with Bulliform phytoliths demonstrating superior predictive capability for water availability. The distinct Si isotopic signatures observed in Bulliform, Elongate, and Stoma suggest that variations in Si fractionation among morphotypes could be linked to differences in Si absorption and deposition processes, likely mediated by water stress.

**Conclusions:**

Our findings align with prior research suggesting that water stress affects Si uptake, potentially altering the Si-water movement relationship. The significant variability in the isotopic data measured indicates the potential involvement of additional environmental, and consequently physiological factors influencing silicon isotope composition in phytoliths, especially Bulliform, which we suggest should be the focus of future research. Our model offers a solid foundation for research in several fields, from agronomic studies aimed at using Si to improve drought-resistance, to palaeoenvironmental and archaeological studies aimed at reconstructing past climate change and human–environment interactions.

## Introduction

Silicon (Si) is a common element in soils, and plant roots are continuously exposed to it. Within plants, Si exists either as soluble silicic acid (H_4_SiO_2_), or as amorphous biosilica (SiO_2_·nH_2_O). The dissolved species is the form in which Si moves in water from the roots to the leaves, while the second represents the solid form, namely phytoliths (Pearsall 2016) or irregularly shaped extracellular deposits. The concentration of Si within plant tissues exhibits a considerable range, spanning from 0.1% up to 10% of the dry weight (Cornelis and Delvaux 2016). This variability is contingent upon numerous factors including genetic and physiological processes, as well as external conditions. Environmental factors exert both direct effects in Si concentration within plants, such as soil Si availability or indirect effect, as for example available water that impacts on transpiration rates and consequently on biosilica transfer and deposition (Cooke and Carey 2023; Zexer et al. 2023).

Silicic acid uptake occurs through root cortical cells, utilising both apoplastic (extracellular space) and symplastic (intracellular space) pathways (Guerriero et al. 2016). Si distribution across plant organs proceeds either via passive diffusion, primarily driven by the transpiration stream (Nawaz et al. 2019), or through an energy-dependent active transport symplastic pathway, relying on transporter channels (Ma et al. 2001). Silicon-accumulating species initially favour passive accumulation until an insufficient Si level stimulates the production of transporters for more substantial active transport (Ma and Yamaji 2008; Katz et al. 2021). Upon initiating the active process, Si concentration within the plant depends on the distribution, density and type of Si transporters (Ma and Yamaji 2006). In rice (*Oryza sativa* L.), four transporter types have been identified: the channel-type Si transporter encoded by Lsi1 or homologous, facilitates Si translocation across the plasma membrane from the apoplast to cells (Deshmukh and Bélanger 2016) through a protein which belongs to a Nod26-like major intrinsic protein (NIP) in the aquaporin family (Ma et al. 2007). Si is transported to proximal apoplastic connections by the efflux transporter Lsi2 (Ma et al. 2006). So far, four Lsi2 homologs are known as Silicon Efflux Transporters (SIET) (Mitani-Ueno et al. 2023). Xylem loading is mediated by the transporter Lsi6 in leaf sheath and blade parenchyma cells (Gaur et al. 2020), offloaded by Lsi3 (Yamaji et al. 2015). The Si translocation pathway based on NIP and proton antiporters proteins has been detected in other cereals such as maize (*Zea mays* L.) (Mitani et al. 2009), and in vegetables such as pumpkin (*Cucurbita pepo* L. Dumort.) (Mitani et al. 2011) and cucumber (*Cucumis sativus* L.) (Sun et al. 2017). In the roots of some grass species, silicon is present in the form of biosilica aggregates, which are arranged to form aggregations in the endodermis of roots, following an ordered array (Zexer et al. 2024). Upon entering the shoots, silicic acid precipitates, forming extracellular silica deposits or phytoliths in the cell wall or within the lumen (Hodson 2019). Once it precipitates it is not thought to be remobilised but there is some evidence of re-translocation among shoot organs (Thorne et al. 2023). Phytoliths can take different shapes (*i.e.*, morphotypes) distinguishable by morphology, size, surface texture and elemental content, depending on the deposition micro-environment (*i.e.*, cell where they form) (International Committee for Phytolith Taxonomy (ICPT) et al. 2019). In accordance with the mechanisms governing the deposition process, two categories of phytoliths have been proposed: cell wall and lumen phytoliths (Hodson 2019). Lumen morphotypes refer to forms resulting from the silicification of the lumen space, such as grasses short cells in the leaf epidermis (*e.g.*, BILOBATE, SADDLE, and CROSS) and Bulliform. Conversely, the deposition of cell wall morphotypes involve silica deposition in the primary and secondary cell wall, such as epidermal Elongate (Piperno 1989).

Prior research has investigated diverse morphotype ratios to elucidate the impact of environmental factors, such as water availability, on phytolith deposition (Miller Rosen and Weiner 1994; Bremond et al. 2005; Madella et al. 2009; Weisskopf et al. 2015; Jenkins et al. 2016, 2020; D’Agostini et al. 2023). More recently, stable silicon isotope values (δ^30^Si and δ^29^Si) in phytoliths emerged as potential proxies for environmental conditions. δ^30^Si in phytoliths has been identified as a proxy for soil conditions, particularly weathering and organic matter content (Leng et al. 2009). Phytoliths have lower δ^30^Si values, thus are preferentially enriched in ^28^Si, than silicic acid in soils indicating a biochemical Si fractionation associated with silicic acid uptake by plants (Ding et al. 2008). Moreover phytoliths become increasingly enriched in ^30^Si up to approximately + 6.5‰ along the plant, from the roots to the inflorescence, as the available silicic acid pool becomes depleted in ^28^Si (Opfergelt et al. 2006a; Sun et al. 2008; Frick et al. 2020a). This enrichment is attributed to equilibrium effects (Stamm et al. 2020) and kinetic effects (Rayleigh fractionation) (Geilert et al. 2014; Poitrasson and d’Abzac 2017), which explain the accumulation of heavy isotopes in the upper parts of the plant. Some uncertainties regarding the type and extent of Si fractionation consequent to the passage through silicon transporters (Lsi1, Lsi2, Lsi3, Lsi6) and potential unidentified homologs (Köster et al. 2009) exist. Despite that, Frick and colleagues (Frick et al. 2020b) argued that whether Si is absorbed through Si permeable channels or passively via water flow, both pathways favour light Si isotopes owing to their higher diffusion coefficient (argument sustained also in Sun et al. 2008).

In archaeology phytolith analysis has been widely employed to identify major and minor domesticated crops (*e.g.*, Piperno and Pearsall 1998; Iriarte 2003; Piperno 2006; Ball et al. 2016a, b), for reconstructing crop-processing activities and use (*e.g.*, García-Granero et al. 2018; Rashid et al. 2019), spatial organisation (*e.g.*, Ball et al. 2016b), and researching ancient plant domestication (*e.g.*, Harvey and Fuller 2005; Cabanes et al. 2011; Madella et al. 2016). Additionally, phytolith assemblages can provide insights into field extensions (Blinnikov et al. 2013) and composition, and past environments (Bremond et al. 2005). Experimental studies have explored the potential of phytoliths as indicators of past water management (*e.g.*, D’Agostini et al. 2023). However, the application of phytoliths as a proxy of water availability in archaeological contexts remains a significant challenge due to the large inter- and intra-specific discrepancies observed, with no consistent results currently available. Given these premises, the primary research question addressed in this study is whether plants that suffered water stress exhibit observable differences in phytolith stable isotope values compared to those grown under well-watered conditions. If water scarcity induces a physiological response observable in Si absorption and deposition that influence isotopic ratio, this could potentially serve as a proxy of water availability in paleoenvironmental and archaeological studies. Moreover, this can have significant implications for research exploring the potential benefits of silicon in enhancing drought tolerance in crops.

**Fig. 1:**
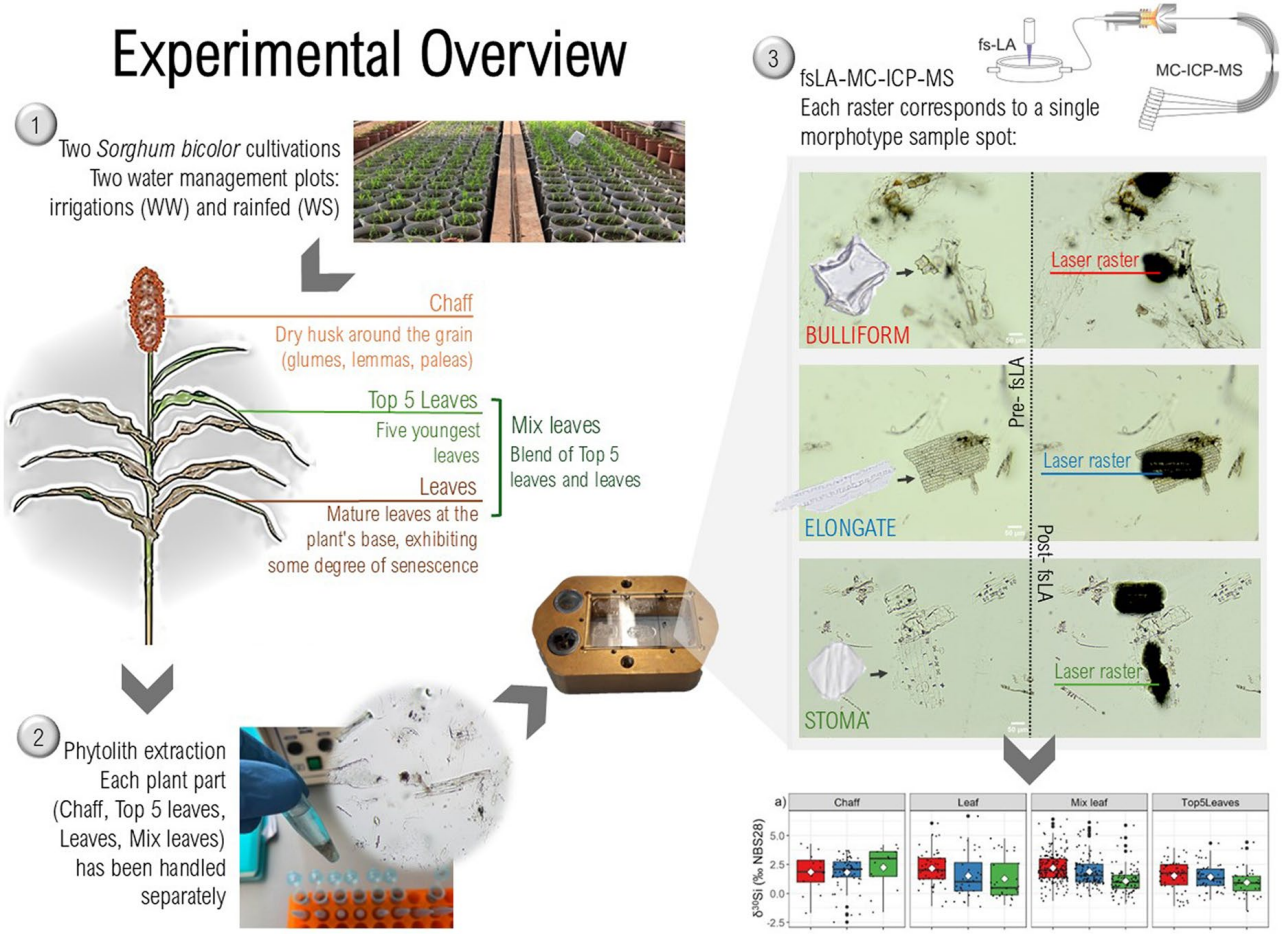
Analysis orientation figure step by step. Step 1: Two cultivation replicas (2019 and 2020) have been conducted in ICRISAT research centre. For each season two water management strategies have been put in place: irrigation, which served as control (well-watered, WW), and water stress, mimicking water scarcity in arid environments (water scarcity, WS). From each plant under analysis three different plant parts were processed separately for phytolith extraction: “Leaves”, “Top 5 leaves”, “Mix leaves” and “Chaff” (Table 2). “Leaves” refers to mature leaves situated at the plant's base, exhibiting some degree of senescence. “Top 5 leaves” denote the five youngest leaves located at the upper apex of the stem. “Mix leaves” represent a blend of these two leaf categories. “Chaff” encompasses the inflorescence husk surrounding the grains. Step 2: Phytolith extractions have been carried out following D’Agostini et al. (2022a) to reduce plant tissue into pure biosilica powder. In Fig. 1 Step 2 biosilica powder in distilled water is shown before drying. Step 3: The extracted biosilica was mounted on a poly-methyl methacrylate (PMMA) microscope slide and then inserted on the fsLA-MC-ICP-MS. The fsLA-MC-ICP-MS is connected to an optical microscope and allows for precise slide scanning and phytolith classification to set the specific laser beam raster over the morphotype of interest (Bulliform, Elongate and Stoma). In Fig. 1 Step 3 is shown how biosilica powder under the optical microscope looks like before and after the laser scanning

To investigate whether Si absorption is influenced by water stress, we examine the stable silicon isotope ratio composition, expressed as δ^30^Si and δ^29^Si of lumen and cell wall morphotypes from different plant parts of *Sorghum bicolor* L. Moench, experimentally grown in a controlled environment (Fig. 1). We focused on different morphotypes because, in principle, Si fractionation should depend on two simultaneous factors: 1) the plant part under examination and its height relative to the roots due to Rayleigh fractionation, and 2) water availability, which influences directly the quantity of Si (and thus the availability during the growth and maturation time of light silicon isotopes), and indirectly the preferential transport of lighter Si isotopes through activated transporters in response to water stress. Based on this hypotheses, we analysed δ^30^Si and δ^29^Si in three different morphotypes: Bulliform, Elongate and Stoma. Although these morphotypes are all found in the plant epidermis, the final tissue reached by silicic acid flow, the relative living cells serve distinct functions and occupy different positions within the leaf tissue. This might result in potential variations in discrimination depending on the involvement of different Si transporters in biosilica relocation and deposition (Cook and Carey 2023; Mitani-Ueno et al. 2023). Additionally, one morphotype originates in the cell lumen (Bulliform) whereas the other two in different regions of the cell wall (Elongate and possibly Stoma) (Hodson 2019), implying diverse selective processes before deposition and potential alterations in δ^30^Si.

Sorghum was chosen as a C_4_ species characterised by active Si transport (Tubaña and Heckman 2015; D’Agostini et al. 2022b). Variations in deposited biosilica in this species are expected to be governed by an energy-dependent physiological response to water scarcity conditions (Sangster and Parry 1971). Sorghum has been examined for its Si absorption capacity: Lsi1 protein sequence analysis by Vatansever et al. (2017) identified two homologues genes encoding transporter channels. A more recent phylogenetic analysis suggested the presence of 5 Lsi2 homologous in sorghum (Coskun et al. 2021)*.* A unique amino acid composition protein involved in silica precipitation in silica cells (Slp1) has been discovered in sorghum (Kumar et al. 2017, 2020). The sorghum varieties selected for the experimental cultivation are landraces, chosen to evaluate the range of phytolith production in “unimproved” crops. This avoids recent breeding modifications that might influence biosilica accumulation or transpiration efficiency, ensuring that the results remain relevant for archaeological interpretations.

## Materials and methods

### Landraces selection

To capture the full intra-species variability potential, we choose varieties from the three distinct ecotypes of drylands, which were also the zones of origin for the domestication of this species: arid (Sudan), semi-arid (Pakistan) and dry sub-humid (Ethiopia) (Manning et al. 2011; Manning and Timpson 2014). Accordingly, we selected seedbank entries based on temperature, precipitation, and humidity data of their area of origin. The climatic data were sourced from the high-resolution datasets provided by the Climatic Research Unit TS3.10 Dataset (Harris et al. 2013) and https://en.climate-data.org (accessed on 15 October 2018) (Table 1). These selections were drawn from the available collection at the ICRISAT genebank, prioritising FAO in-trust varieties (varieties equipped with crop passport descriptors, collected and recorded in agreement with the country of origin and local communities).

**Table 1:** Selected landraces from ICRISAT genebank used for the experimental cultivation indicated with their accession number. Data are specific to the location of origin of the grains. The two Pakistani and Ethiopian varieties were recollected in the same region. Mean temperature, average sun hours and humidity and are expressed as the annual mean of the site location. Precipitation and rainy days represent the total annual condition. Weather data was collected between 1991–2021 for temperature, precipitation, humidity, rainy days. Sunhours use the timeframe 1999–2019

Climatic conditions of Sorghum landrace origins			
	Sudan	Pakistan	Ethiopia
***Mean temperature*** ***Average sun hours*** ***Precipitation*** ***Rainy days*** ***Humidity***	32.79 ºC 10.9 h 70 mm 13 days 25.16%	27.34 ºC 10.7 h 152 mm 15 days 44.66%	27.63 ºC 10.5 h 519 mm 60 days 37.16%
**Sorghum landraces**	IS23075	IS35216 IS35217	IS11060 IS11061

### Experimental cultivation

The experimental cultivation was carried out at the ICRISAT research centre, located in the semi-arid tropic province of Hyderabad, through two replicas cultivated between February and May of 2019 and 2020. Lysimeters were used for the experimental cultivation due to their ability to control plant water availability while monitoring individual plant transpiration. These PVC tubes (200 cm in length and 25 cm in diameter) were installed next to each other into two parallel pits to mimic natural field conditions, including plant spacing (11 plants per square metre), soil depth for groundwater exploration (tubes of 2 m by 25 cm diameter of soil availability per plant in), and general outdoor growing conditions (but protected by a rain-out shelter in case of rainfall). Lysimeters were filled with a mixture of 1:1 Alfisol-Vertisol agricultural soil because previous evidence indicates that Si absorption in commercial soil or in hydroponics is not comparable to that in natural soil (Keeping 2017). Further details on the experimental setup can be found in D’Agostini et al. 2022a, b and D’Agostini et al. 2023, 2024, following the methodology outlined in Vadez et al. (2011).

In both replicas (2019 and 2020), we tested two water management strategies: (a) irrigation, which served as control (well-watered, WW), and (b) rainfed conditions, mimicking water scarcity in arid environments (water scarcity, WS). WW plants were watered weekly to maintain soil field capacity at 80%, optimal for crops, such as sorghum, adapted to dry climates (Zaman-Allah et al. 2011). In WS conditions, the aim was to replicate a real rainfed scenario where water availability diminishes during the reproductive stage (Portmann et al. 2010). Therefore, WS plants were watered similarly to WW plants until flowering, after which irrigation ceased until maturity. Total water provided to WS plants did not exceed 300 mL. Transpiration curves confirmed intense water stress in WS plants, rapidly decreasing to 10% in respect to WW replicates, reaching cuticular transpiration. For each of the two treatments, five replicates of each landrace were cultivated, randomly distributed within the cultivation plot. Weekly water inputs and transpiration values for each replicate in both cultivation conditions are detailed in file S1.

### Soil analysis

Soil Si content in the lysimeters was analysed to exclude any discrepancies arising from differences in soil Si availability between replicates. Soil samples were taken in five cylinders (two WW and three WS) randomly chosen after the 2020 harvesting campaign. The soil analysis comprises a total of 40 samples, four cylinders were sampled from the top soil every 10 cm down to 90 cm deep, whereas the last WS cylinder was only analysed in depth (from 60 to 90 cm) to understand the Si component at the root apex level (n = 9 samples per cylinder, n × 4 cylinders = 36 + n/2 (half cylinder) × 1 = 4, total = 40 soil samples). Soil elemental composition was analysed at the Barcelona Institute of Geosciencies of the Spanish National Research Council (GEO3BCN-CSIC) using a pXRF spectrometer (Bruker Tracer-5 g) with mudrock calibration on samples analysed for 120 s. The average soil silicon [Si] content (± 1 standard deviation) across cylinders is summarised in Table 2. Based on these results, the silicon [Si] content is considered consistent across all samples. Further information on the elemental content of the soil can be found in file S1.

**Table 2:** Soil silicon [Si] content of the 40 samples analysed, divided by water treatment

	Silica content in lysimetrers					
		n	Mean ± 1 standard deviation % (w/w)	Median % (w/w)	Min % (w/w)	Max % (w/w)
**All cylinders**		40	8.90 ± 1.85	9.63	3.71	11.19
**WW cylinders**		27	9.41 ± 1.60	10.07	6.10	11.19
**WS cylinders**		13	8.48 ± 1.97	9.31	3.71	10.76

### Phytolith preparation for laser ablation

We developed a method that ensures the extraction of nearly pure biosilica powder in different cereals plant parts, maximising the preservation of silica skeletons (crucial targets in fsLA analysis) and prioritising the use of chemicals that do not alter the isotopic results. The full protocol is available in D’Agostini et al. (2022a) and combines dry ashing and wet oxidation. We carried out phytolith extractions at the Laboratory of Environmental Archaeology at University Pompeu Fabra (Barcelona, Spain) on a selected number of replicates to achieve statistical significance while minimising extraction and analytical time. One replicate for each landrace grown under WW and WS conditions was selected and for each replicate, three different types of plant part were processed separately for phytolith extraction: “Leaves”, “Top 5 leaves”, “Mix leaves” and “Chaff” (Table 2). “Leaves” refers to mature leaves situated at the plant's base, exhibiting some degree of senescence. “Top 5 leaves” denote the five youngest leaves located at the upper apex of the stem. “Mix leaves” represent a blend of these two leaf categories. “Chaff” encompasses the inflorescence husk surrounding the grains.

The extracted biosilica was mounted on a poly-methyl methacrylate (PMMA) microscope slide using Entellan New® as a mounting medium. Entellan New® facilitates clear visualisation of phytoliths, aiding in their classification for laser pathway selection. Prior to sample analysis, Entellan New® underwent testing for blank contamination to ensure the absence of any interference. A drop of Entellan New® was initially applied to the slide, followed by the application of the biosilica powder over it using a clean spatula. This method prevented superimposition of phytoliths, facilitating laser penetration without encountering excessively thick Entellan New® layers, which could diminish the signal.

### Laser ablation

Stable silicon isotope ratio analysis on Bulliform, Elongate and Stoma were conducted using femtosecond laser ablation multi-collector inductively coupled plasma-mass spectrometry (fsLA-MC-ICP-MS, fsLA hereafter), at the German Research Centre for Geosciences Potsdam in the Helmholtz Laboratory for the Geochemistry of the Earth Surface (HELGES). fsLA was selected as it allows the analysis of the isotopic composition of individual phytoliths of sufficient thickness or length (Frick et al. 2016, 2019). Connected to an optical microscope, the machine allows for precise laser beam scanning, approximately 25 µm in diameter, directly onto the sample (Fig. 1). The laser path is adapted to each single phytolith surface, thus preventing the inclusion of non-siliceous particles in the background. Furthermore, the nearly athermal nature of fsLA ensures the accuracy of LA-ICP-MS performance, minimising matrix effects and isobaric interferences in thick samples, enabling the analysis of heterogeneous low mass materials like phytoliths (Poitrasson and d’Abzac 2017). In situ fsLA analyses were conducted using a custom-built deep-UV (196 nm) femtosecond laser ablation system (see Schuessler and von Blanckenburg 2014 for technical details) coupled with an inductively plasma multicollector mass spectrometer (Thermo Fisher Scientific Neptune MC-ICP-MS, equipped with the Neptune Plus Jet Interface). To maintain a balance between the required small spot size for analysing individual phytoliths and the need to keep constant laser intensity to prevent crater depth enlargement, a small-size raster (50–100 µm) was utilised at a fast scan speed (40 µm*s^−1^) with a variable laser pulse repetition rate (frequency 20–40 Hz) (Frick et al. 2019). The Si isotope results are reported in the δ-notation as permil deviation relative to NBS28 (Coplen et al. 2002).

$$\delta^{30}{Si/}^{28}Si\equiv\delta^{30}Si\equiv\left[\frac{\left(\frac{\delta^{30}Si}{\delta^{28}Si}\right)sample}{\left(\frac{\delta^{30}Si}{\delta^{28}Si}\right)NBS28}-1\right]\left[in\;\permille\right]$$

$$\delta^{29}{Si/}^{28}Si\equiv\delta^{29}Si\equiv\left[\frac{\left(\frac{\delta^{29}Si}{\delta^{28}Si}\right)sample}{\left(\frac{\delta^{29}Si}{\delta^{28}Si}\right)NBS28}-1\right]\left[in\;\permille\right]$$

Quality control standards and uncertainty assessment were adhered to by alternating samples evaluation with measurements of standard material NBS28 (quartz sand, δ^29^Si $\equiv$ 0 ‰ and δ^30^Si $\equiv$ 0 ‰) and of secondary reference materials such as ATHO-G, BHVO 2G, and GOR132-G (basalt glasses) (Jochum et al. 2005; Schuessler and von Blanckenburg 2014). For more information on the experimental configuration see Frick et al. 2019. All δ^30^Si and δ^29^Si results are accompanied with uncertainties at the 95% confidence level. The variance is obtained by calculating the standard deviation of Si isotope ratio integration cycles acquired during < 3 s of analysis (Frick et al. 2019) and expressed as the standard deviation of the mean derived from Student’s t distribution at 95% confidence. Acceptable results, apart from producing a mass bias drift between the two bracketing calibrators of < 0.30‰ in ^30^Si/28Si, must follow the mass-dependent terrestrial fractionation line in a three-isotope plot of δ30Si and δ^29^Si within analytical uncertainties (Frick et al. 2019). All the measurements reported in this study met these criteria while all data that did fall outside of these criteria were excluded (Table 3).

**Table 3:** Final number of samples included in the analysis per year of experimental cultivation

Sample count						
	2019			2020		
Sorghum landrace	WW	WS	Total	WW	WS	Total
***S2***	***Mix leaves*** Bulliform 14 Elongate 10 Stoma 11	***Mix leaves*** Bulliform 19 Elongate 7 Stoma 7	***68*** 33 17 18	/	/	
***S4***	***Mix leaves*** Bulliform 17 Elongate 11 Stoma 17	***Mix leaves*** Bulliform 17 Elongate 7 Stoma 2	***71*** 34 18 19	/	/	
***S5***	***Mix leaves*** Bulliform 20 Elongate 8 Stoma 8	***Mix leaves*** Bulliform 20 Elongate 10 Stoma 11	***77*** 40 18 19	/	/	
***S9***	***Mix leaves*** Bulliform 19 Elongate 13 Stoma 16	***Mix leaves*** Bulliform 19 Elongate 19 Stoma 11	***97*** 38 32 27	***Chaff*** Bulliform 11 Elongate 16 Stoma 6 ***Leaves*** Bulliform 13 Elongate 10 Stoma 9 ***Top 5 leaves*** Bulliform 18 Elongate 15 Stoma 8	***Chaff*** Bulliform 5 Elongate 14 Stoma 6 ***Leaves*** Bulliform 10 Elongate 9 Stoma 10 ***Top 5 Leaves*** Bulliform 18 Elongate 12 Stoma 9	***58*** 16 30 12 ***61*** 23 19 19 ***80*** 36 27 17
***S10***	***Mix leaves*** Bulliform 8 Elongate 8 Stoma 12	***Mix leaves*** Bulliform 17 Elongate 12 Stoma 7	***64*** 25 20 19	***Chaff*** Bulliform NA Elongate 16 Stoma 1 ***Leaves*** Bulliform 14 Elongate 5 Stoma 6 ***Top 5 leaves*** Bulliform 17 Elongate 15 Stoma 16	***Chaff*** Bulliform NA Elongate 12 Stoma NA ***Leaves*** Bulliform 13 Elongate 4 Stoma 8 ***Top 5 leaves*** Bulliform 3 Elongate 5 Stoma 7	***29*** NA 28 1 ***50*** 27 9 14 ***63*** 20 20 23

For each sample 20 Bulliform, 20 Elongate and 20 Stoma were analysed for a total of 1320 phytoliths (Table 2). Each laser raster corresponded to either a single Bulliform, Elongate or Stoma or to multiple cells of the same morphotype, either included in a silica skeleton or as part of a phytolith cluster. Classification parameters and definitions of each morphotype, according to the International Code of Phytolith Nomenclature 2.0 (ICPN 2019), and reference pictures are available in file S1. For total phytolith concentration, the remaining morphotypes present in each plant part, and reference photos, refer to D'Agostini et al. 2024, Supplementary Material File S2. – Phytolith Data. Other morphotypes such as grass short cells were excluded from this analysis for multiple reasons: mainly technical and experimental constraints due to their small size and the unclear mechanisms governing their silicon deposition.

### Statistical analysis

Descriptive statistics were calculated for the biosilica content (g) and the silicon isotopic composition of phytoliths in different plant parts and water treatments to assess sample distributions. Mann–Whitney tests have been used to determine significant differences between the WW and WS sample groups, with the confidence level set at 95%.

#### Predicting water availability

A logistic regression model, implemented using the generalised linear model (glm) function, was employed to evaluate whether the silicon isotopic composition of phytoliths could predict water treatment categories (WW *versus* WS). δ^30^Si has been tested as a predictor variable. The logistic regression model is expressed using the following probability distribution formula:

$$p(x) = \frac{1}{1 + e{}^{-({\beta 0 +\beta 1x)}}}$$

p(x): represents the probability of a specific silicon isotope value is the effect of WW conditions; β0: corresponds to the intercept estimate value generated once the model has been executed; β1: corresponds to the estimate of the isotope value of interest and x: corresponds to the isotope value to implement into the formula to estimate the probability it comes from a plant grew in WW condition.

The model's performance was assessed using a confusion matrix and various performance metrics, including accuracy, precision, recall, and F1-score, to evaluate the predictive efficacy of different morphotype isotopic compositions and isotope ratios. Additionally, confidence intervals for the predictions and the accuracy of these intervals were computed to further validate the model's reliability. We employed a data splitting and model validation approach to evaluate the performance of our predictive model. The dataset was randomly divided into a training set (80% of the data) and a testing set (20% of the data) while ensuring a homogeneous distribution of variables through the stratified sampling method. The training set was used to fit a logistic regression model, which included the interaction terms among the predictor variables. The fitted model was then used to predict outcomes on the testing set. To assess the model's performance, we compared the predicted outcomes to the actual outcomes in the testing set using a confusion matrix.

Statistical analyses were executed in R (version 4.3.1) using the packages caret (6.0 −94), dplyr (1.1.4), ggplot2 (version 3.4.3), ggpubr (version 0.6.0), MASS (version 7.3 −60) and tidyverse (version 2.0.0). Scripts are available in file S2.

## Results

The results of the in situ phytolith analysis using fsLA-MC-ICP-MS are summarised in Tables 4 and 5, while all individual results are available in the Online Resources: Table S1-S2. Both the amount of extracted biogenic silica and the silicon isotope composition proved to be heterogeneous across the different plant parts analysed and among the various morphotypes. Bulliform morphotypes have shown a good performance in predicting water availability.

**Table 4:** Mean and 1 standard deviation of total extracted biosilica in dry plant part (each sample weighing 0.1 g, percentages by weight are in the adjacent column) and of δ^30^Si along with the median and their corresponding *p*- values derived from the Mann–Whitney test

	Biosilica and δ3⁰Si basic statistics									
			n	Si % (w/w)	Biosilica (g) ± σ	*p*	δ^30^Si mean (‰) ± σ	δ^30^Si max and min (‰)	δ^30^Si median (‰)	*p*
Chaff (2020)		WW	50	15.1%	0.0156 ± 0.0004	** < 0.001**	2.12 ± 1.31	Max 4.29 Min −1.69	2.28	0.11
		WS	37	8.1%	0.0084 ± 0.0016		1.58 ± 1.67	Max 4.21 Min −2.47	1.75	
Top 5 leaves (2020)		WW	89	10%	0.0103 ± 0.0014	** < 0.001**	1.07 ± 1.13	Max 4.42 Min −1.51	0.96	** < 0.001**
		WS	53	10.6%	0.0107 ± 0.0037		1.78 ± 1.18	Max 4.34 Min −1.03	1.71	
Mix Leaves (2019)		WW	191	9.1%	0.0093 ± 0.0018	** < 0.001**	1.40 ± 0.99	Max 5.12 Min −0.84	1.34	** < 0.001**
		WS	186	8.5%	0.0087 ± 0.0015		2.26 ± 1.47	Max 6.40 Min −0.91	2.00	
Leaves (2020)		WW	57	5.3%	0.0055 ± 0.0012	** < 0.001**	2.20 ± 2.02	Max 6.67 Min −1.14	2.05	**0.01**
		WS	54	9.5%	0.0098 ± 0.0027		1.26 ± 1.17	Max 3.52 Min −1.01	1.02	

*n* corresponds to the number of samples examined for each category. Significant *p*-values at *p* < 0.05 are in bold

**Table 5:** Mean and 1 standard deviation of δ^30^Si along with the median and their corresponding *p*- values derived from the Mann–Whitney test

δ^30^Si statistics							
	Plant part	n	δ^30^Si mean (‰) ± σ		δ^30^Si median (‰)		*p*
			WS	WW	WS	WW	
Bulliform	Leaves (2020)	50	2.20 ± 0.90	2.13 ± 1.85	2.12	1.82	** < 0.001**
	Mix leaves (2019)	172	2.68 ± 1.02	1.67 ± 1.27	2.61	1.62	**0.003**
	Top 5 leaves (2020)	55	2.03 ± 1.33	1.24 ± 1.18	2.30	1.46	** < 0.001**
	Chaff (2020)	16	2.28 ± 1.61	1.65 ± 1.70	2.63	1.79	** < 0.001**
Elongate	Leaves (2020)	32	0.72 ± 0.79	2.43 ± 2.40	0.63	3.19	** < 0.001**
	Mix leaves (2019)	108	2.31 ± 1.61	1.45 ± 0.71	1.87	1.29	**0.02**
	Top 5 leaves (2020)	48	1.98 ± 1.28	1.12 ± 0.83	2.19	1.03	**0.01**
	Chaff (2020)	58	1.21 ± 1.56	2.32 ± 0.93	1.60	2.41	** < 0.001**
Stoma	Leaves (2020)	29	0.44 ± 0.80	2.11 ± 2.01	0.23	2.58	** < 0.001**
	Mix leaves (2019)	97	1.18 ± 1.19	1.00 ± 1.03	1.04	0.85	0.07
	Top 5 leaves (2020)	39	1.25 ± 0.64	0.73 ± 1.34	1.16	0.45	0.49
	Chaff (2020)	13	2.59 ± 1.85	1.95 ± 2.00	3.03	2.65	** < 0.001**

**Fig. 2:**
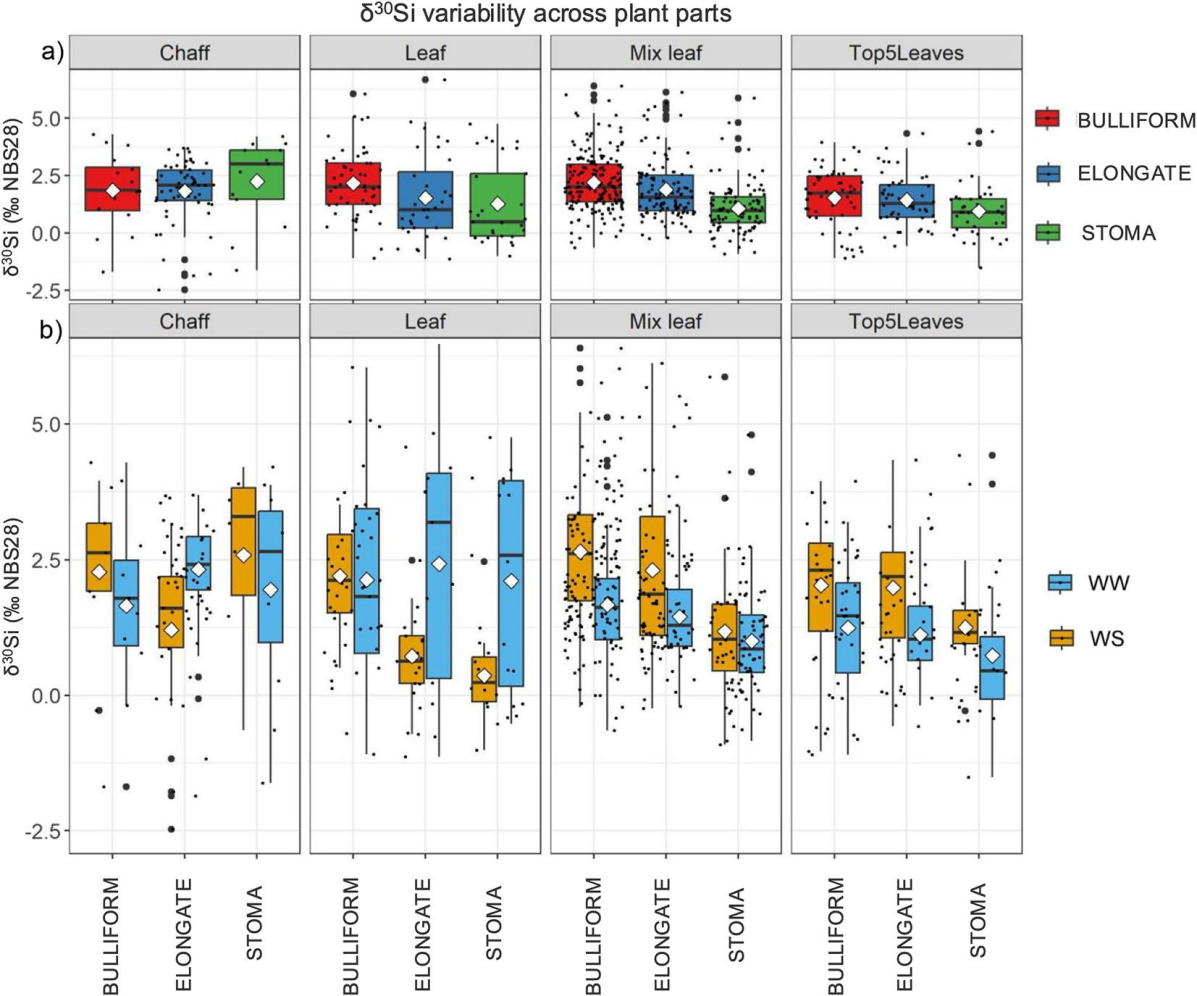
δ^30^Si variability across plant parts. The mean, median, and standard deviation of Si are represented through boxplots, organized by plant part and: **a**) by morphotype **b**) by morphotype and water treatment. Black dots represent individual samples, thicker ones indicate outliers, and the white diamond represents the mean. The specific values are presented in Table 5

*n* corresponds to the number of samples examined for each category. Significant *p*-values at *p* < 0.05 are in bold. The graphical representation of this table is shown in Fig. 2

### Data exploration

The biosilica content and its isotopic values are comparable across the two years of experimentation, with variations independent of the experimental year (*p*-value = 0.1164 for δ^29^Si; *p*-value = 0.1442 for δ^30^Si between 2019 and 2020). The accumulated biosilica in the leaves ranges between 5 and 10% of the dry weight, while the chaff has slightly higher values, reaching a maximum of 15% of the dry weight. These values fall within the range for Poaceae (high silica accumulators) (Hodson et al., 2005; Tubaña and Heckman 2015). On average, δ^30^Si values range from −2.47‰ to + 6.67‰. The heaviest results have been observed in Elongate WW Leaves, while the lightest values were found in Elongate WS Chaff. These data are generally in line with previous studies (Ding et al. 2005, 2008; Opfergelt et al. 2006b; Hodson et al. 2008; Sun et al. 2008; Prentice and Webb 2016; Frick et al. 2019), although the upper limit is higher than previously reported (+ 6.1‰).

First, the results from our study show that the water treatment affects overall biosilica accumulation. Differences are evident in each of the analysed plant parts (Table 5), although the relationship varies. The WW chaff accumulated an average of 15.1% biosilica compared to 8.1% in WS, whereas leaves accumulated an average of 5.2% in WW and 9.5% in WS. Secondly, water availability also affects phytolith isotopic composition. The δ^30^Si values in leaves were significantly different between WW and WS (Table 4), with the largest differences observed in Top 5 Leaves. It is worth noting that the correlation between δ^30^Si and biosilica accumulation is not always negative, indicating variability among plant parts. Chaff WW samples have on average, more biosilica than their WS counterparts (Table 4), but the δ^30^Si is higher in WW than in WS. Conversely, WS Leaves have more biosilica than WW leaves, but on average, the δ^30^Si is higher in WW.

The variability within the single plant parts is high (Fig. 2), with the highest standard deviation found in WW Leaves, averaging around 2.02‰, while the lowest in the WW Mix Leaves at 0.99‰. However, when analysing each morphotype individually, the variability decreases (Table 4). For example, Bulliform values below 0.5‰ are found exclusively in WS replicates. In Chaff Elongate (diagnostic for sorghum), values below 1.5‰ occur only in WW conditions, whereas values above 1.5‰ in Leaves Elongate are observed solely in WS conditions. Leaves Stoma values above 1.5‰ are characteristic of WS conditions (Fig. 2). ^30^Si enrichment curves along the plant are not constant and homogeneous and are closely related to the type of morphotype being examined (Fig. 2). Only WS Stoma exhibit an enrichment rate increasing up the plant (Leaves < Mix Leaves < Top 5 Leaves < Chaff). Elongate show low δ^30^Si values in WS Leaves and particularly high values in WW Leaves.

The greatest variability was observed in the Elongate (∆30Si = 2.43‰, min = −1.14‰, max = 6.67‰) and Stoma (∆30Si = 2.11‰, min = −0.52‰, max = 4.75‰) of senescent leaves under WW conditions. Interestingly, variability decreases when different sorghum varieties are considered separately. For instance, the landraces S9 and S10, both varieties are from Pakistan, exhibit the lowest and highest discrimination values for δ^30^Si, indicating a certain"sensitivity"in silicon isotope discrimination (see Online Resources Table S6-S7-S8-S9 and individual results on file S1).

### Predicting water availability

There is a relationship between watering regimes (WW *versus* WS) that is more evident at the morphotype level rather than at the plant part level (Table 5). Bulliform and Elongate show different δ^30^Si values between WW and WS in all examined plant parts, indicating their potential to accurately predict water availability (subjected to the Mann–Whitney test, consistently show *p* < 0.05 between WW and WS). Conversely, for Stoma, significant differences in δ^30^Si between WW and WS are only observed in the Chaff and Leaves, but not in young, photosynthetically active tissues Top 5 Leaves.

**Fig. 3:**
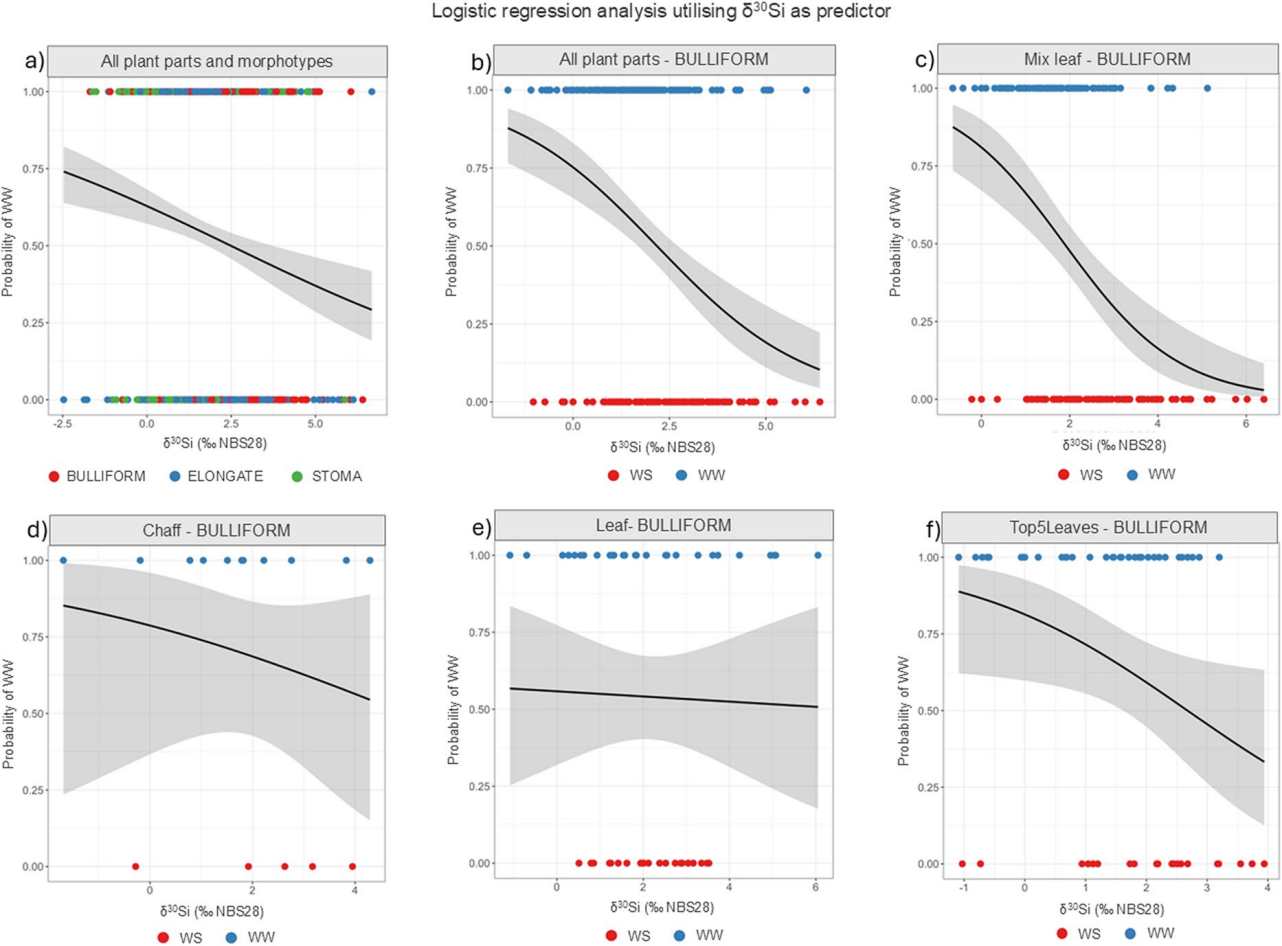
Logistic regression analysis output utilising δ^30^Si as predictor. The x-axis represents increasing δ^30^Si values, while the y-axis denotes predictive model outputs: 1 signifies 100% probability of WW, 0 denotes 0% probability of WW, and 0.50 corresponds to a 50% probability of WW (and WS as logic consequence). Shaded grey areas indicate the 95% confidence intervals. **a**) All morphotypes from all plant parts considered together: **b**) Only Bulliform from all plant parts; **c**) Only Bulliform from mixed leaves; **d**) Only Bulliform from chaff (not abundant in chaff); **e**) Only Bulliform from leaves; **f**) Bulliform cells from the top 5 leaves

To test the efficiency of each morphotype in indicating the watering status under which the plant grew, we applied logistic regression using δ^30^Si as predictor (column “Predictors” Table 6), while keeping the morphotypes separate. The results for each model are shown in Table 5, while the 95% confidence intervals, metrics and model validation are available in the online resources (Table S4). The graphical representation of the model is provided in Fig. 3, which shows the predictive sigmoidal curve for the three morphotypes considered together (Fig. 3a) and only the Bulliform from each plant part Fig. 3b, c, d, e and f). Table 6 show that δ^30^Si is an effective predictor when measured in Bulliform but not in Elongate or Stoma that are not significant predictors and are unlikely to be useful for archaeological and paleoecological reconstruction. For Bulliform, values above approximately 2.5‰ correspond to samples grown under WS conditions (Fig. 3b, c and f), while lower values correspond to WW conditions. Whereas the Bulliform in the chaff (which are few and vestigial) and in the more mature leaves appear to be less indicative when considered separately.

**Table 6:** p-values resulting from the logistic regression model using the glm function, with treatment (WW-WS) as the dependent response variable and dependent predictor variables in the second column of the table. Significant *p*-values at *p* < 0.05 are in bold

Table 6	Predictors	*p*-value
Bulliform	Intercept (β0)	** < 0.001**
	δ^30^Si	**0.04**
Elongate	Intercept (β0)	0.92
	δ^30^Si	0.92
Stoma	Intercept (β0)	**0.02**
	δ^30^Si	0.73

The model developed for Bulliform shows an accuracy of 0.68, sensitivity of 0.63, and specificity of 0.73 for cross validation. The confusion matrix indicates an accuracy of 0.67 and a precision of 0.68. The fact that the fitting and the cross-validation metrics have similar values indicates that the model is not overfitting. These metrics remain around 70% whether considering the landraces separately or together (complete data available in the online resources Tables S4-S5). This consistency is attributed to the high dispersion of isotopic data (see mean, standard deviation, and median in Table 4), which affects the model's predictive accuracy. However, to maintain data integrity, we did not scale the values or remove outliers. Despite this, the model still achieves an accuracy of around 70% even in predicting blind data.

## Discussion

This study was conducted to verify whether plants subjected to water stress (WS) exhibit observable differences in biosilica accumulation and phytolith silicon isotopic composition compared to those grown under well-watered conditions (WW). We sought to understand whether water scarcity induces a physiological response in Si absorption and deposition, which could serve as a proxy. The results show that the Si isotopic composition of sorghum phytoliths is related to the plant watering conditions during growth, however the signal can only be detected by examining individual morphotypes separately for each plant part. Indeed, among the morphotypes analysed, Bulliform types have shown the best performance in predicting water availability.

This study also highlights a wide variability among samples that prevents a clear understanding of the governing mechanisms in Si movement along plants. However, this wide variability (Fig. 2) could be related to several factors which nonetheless help in understanding the phenomenon of biosilica deposition in plants. The intra-specific variability of each landrace in responding to water stress would explain why there is a wide range of δ^30^Si values in S9-S10 of Pakistani origin and less in others. These differences could be due to the fact that some varieties are less affected by water stress, or their genetics related to silicon absorption and deposition are distinct (Lux et al. 2002; Hartley et al. 2015). It is known that, in many species, genetic intra-species differences exist in terms of biosilica accumulation (Katz et al. 2021). Comparisons among different homologs of transporter channels (Lsi1, Lsi2, Lsi3 and Lsi6), especially in their distribution and density, have primarily been conducted on different species (e.g., Coskun et al. 2021), but the intra-specific variability in sorghum or other C_4_ species has never been explored in depth (Markovich et al. 2019, 2022). In our previous study, which aimed to quantify the effect of transpiration on biosilica accumulation in different landraces, we had already highlighted some variability even among different sorghum varieties (D’Agostini et al. 2022b), that appears to be confirmed by the data emerging from this study on the isotopic composition of phytoliths. In addition, other environmental variables with discriminatory effects that we are not currently considering could explain this wide data variability. For example, the micro-temperature and level of humidity during phytolith formation, whose effect has not yet been tested, could be a contributing factor.

Our results align with previous studies that suggest that water stress could impact the quantity of Si absorbed and fractionated by plants by affecting the modulation of transpiration. Cooke and Carey suggest that stress exposure can alter the relationship between Si and water movement in plants (Cooke and Carey 2023). Hence, stress should be considered an important mechanism contributing to the diversity of Si accumulation within and across sorghum plants. Ding et al. (2008) also previously proposed that δ^30^Si and δ^29^Si values should be related to water as Si availability during plant growth. In theory, Si availability for the plant is connected both to the soil moisture level and to the amount of water transpired by the plant, both of which are linked to the level of water availability (De Tombeur et al. 2020). If Si is absorbed along with water, then when the water supply is consistent, there will also be higher absorption of Si. Thus, if the pool of ^28^Si, ^29^Si, and ^30^Si increases, Rayleigh fractionation should lead to greater isotopic discrimination, as the lighter isotopes (^28^Si and ^29^Si) are preferentially removed and precipitate more rapidly, enriching the remaining pool in ^30^Si. This assumption has been supported by the results presented by Opfergelt et al. (2006) regarding the Si absorption and distribution in *Musa acuminata* Colla. Opfergelt and colleagues suggest that the preferential passage of light Si isotopes contributes to progressive isotopic fractionation of the solution moving from uptake sites in roots to the transpiration termini in leaves. Geilert et al. (2014), who conducted a series of flow-through experiments to investigate Si isotope fractionation during abiotic precipitation, supported this theory by concluding that the flow-through system could be an approximate model for a transpiring plant where water is continually flowing through the xylem when temperatures range between 10 °C and 20 °C.

The better efficiency of Bulliform in predicting watering conditions compared to Elongate and Stoma (Table 6 and Fig. 3) could be due to the following reasons:

1. The most likely option is that it is related to the process of biosilica forming phytoliths, which varies in each morphotype and depends on the cell part where precipitation begins: in the cell wall or in the cell lumen (Hodson 2019). This suggests that the analysis of the isotopic composition of morphotypes could have a future in researching the different deposition processes characterising the silicification of different cells.
2. The relative size of the Bulliform morphotype, which is larger and more voluminous compared to the other two morphotypes (International Committee for Phytolith Taxonomy (ICPT) et al., 2019). Cell size has already been suggested for different levels of Si discrimination in sea sponges (Hendry and Robinson 2012; Cassarino et al. 2018), and we suggest, according to our results, that it could make Bulliform more sensitive to environmental changes.
3. The variation in structure, as seen in diatoms (Leng et al. 2009). Differences between morphotype structure have been demonstrated, such as different refractive properties or dissolution rates (Cabanes et al. 2011; Schaller et al. 2014), which could be derived from distinct compositions but also from the time of formation, with varying quantities of trapped H_2_O molecules and surface hydroxyls, leading to different opaline structure (Stamm et al. 2020). However, this has not yet been experimentally verified for specific phytoliths.
4. Even if less likely we cannot completely rule out the possibility of an active role that Bulliform play in response to water stress. Si deposition in Bulliform has been proven to reduce leaf wilting (Amin et al. 2016; Fernández Honaine and Osterrieth 2012) and Bulliform involvement in the structural response to water stress remains highly plausible. However, we are inclined to dismiss this fourth possibility because our data suggest that even the Elongate morphotype plays an active role in water stress response (see discussion in the next paragraph).

We believe it is not only the process of formation itself but also the micro-environment of deposition and the degree of hydration that influence structural heterogeneity, ultimately leading to variations in the distribution of light and heavy silicon isotopes among specific morphotypes. This is evident not only from the predictive model (Table 6 and Fig. 3) but also because the three morphotypes have shown distinct average levels of fractionation in the epidermal tissues of the same leaf (Table 4).

Our research shows that watering can indeed be predicted through the isotopic content of Bulliform. However, it has also highlighted three other important aspects:

1. Under water stress conditions, sorghum does not necessarily deposit less biosilica. Depending on the plant part examined, there could be more biosilica compared to the respective WW replicas, as biosilica may serve a function under water stress conditions and its absorption may be induced by drought (Table 4).
2. Lower percentages of biosilica do not correspond to higher δ^30^Si values, indicating that discrimination is not solely and exclusively dependent on the amount of Si available (Table 4).
3. There is significant variability in δ^30^Si values among plant parts, and these values do not necessarily increase along the water stream (from Leaves < Mix Leaves < Top 5 Leaves < Chaff).

Assuming that, on a mass basis, lighter isotopes ^28^Si and ^29^Si reacts faster than the heavier ^30^Si during the entire process of biosilica transport along the plant at each step (an assumption still to be fully experimentally verified), but recognizing also that if bonds such as Si–O were to be broken, ^30^Si could be preferred due to its stronger bond strength, these three phenomena mentioned earlier, can be explained by different factors that might have simultaneously contributed. First, the tissue age and temporal variation in phytolith deposition depending on the plant phase. Tissue age would explain why, on average, the Top 5 Leaves always have lower δ^30^Si values compared to the Chaff and also the Leaves (Fig. 2 and Table 5) and it has already been suggested as a possible source of biosilica variation (Motomura 2004). Secondly, the role of specific morphotypes both in water stress response or in specific moment of plant life cycle: photosynthetically active leaves do not deposit biosilica in Stomata to avoid blocking them (Kumar et al. 2017), but active leaves might deposit it to form Elongate to increase leaf brightness and protect the surface against pathogens (Goto et al. 2003). This would explain a majority of relative values in Table 5: for example, similar δ^30^Si values in Leaves WW for all morphotypes indicating likely simultaneous formation, while much lower δ^30^Si values for Elongate WS indicating possible earlier formation. Cooke and Carey (2023) noted that when Si was added to unstressed (control) plants, there was no significant impact or consistent pattern on transpiration or Stomatal conductance. However, when Si was added to stressed plants, they observed significant increases in water movement, including both transpiration and conductance (Cooke and Carey 2023). This would confirm not only a relationship between water availability and/or stress and biosilica but also that specific morphotypes are an active response to water stress. Lastly, the concept of biosilica translocation/relocalization should be taken into consideration: the great variability in δ^30^Si values might be due to the fact that even if tissues form simultaneously, phytoliths may form at different times following paths of biosilica relocalization for specific defence or structural reasons (Thorne et al. 2023). In this study, Stoma and Elongate in WS leaves have low δ^30^Si values, showing an early formation that could be also a consequence of the relocalization of biosilica. This assuming that Stoma and Elongate prevent leaf curling or tissue collapse weakened by water stress and block water loss through transpiration by closing many Stomata with phytolith formation (Rodrigues et al. 2003; Meunier et al. 2017; Gao et al. 2020). The theory is not without merit because it has been proposed that Si can improve water balance in plants, especially when deposition occurs at the epithelial/surface level as in Elongate and Stoma. Si can increase cuticle thickness (Hattori et al. 2005) and membrane stability (Agarie et al. 1998), or deposit in the cuticular layer (albeit predominantly recorded in rice, see Yoshida et al. 1962), reducing transpiration as a way to conserve water use. Together, these physical changes often increase the water potential in stressed plants supplied with Si (Gong and Chen 2012).

## Conclusion

This study sheds light on the complex interactions between water availability and silicon (Si) dynamics in plants, specifically focusing on the isotopic composition of Si in sorghum phytoliths under varying water conditions. Our findings confirm that water stress (WS) triggers significant physiological responses in Si absorption and deposition. The isotopic data revealed that differences in δ^30^Si values are linked to water stress, particularly when examining individual phytolith morphotypes. Among these, Bulliform morphotypes showed a stronger correlation with watering conditions, making them promising indicators of environmental water availability. This insight highlights the potential of using Bulliform phytoliths in paleoenvironmental and archaeological studies to infer historical water conditions, offering a new proxy for understanding ancient climates and ecosystems. It also stresses the importance of including phytolith analysis in plant physiology studies, especially those related to improving drought tolerance in crops.

Moreover, the study's results emphasise the diversity and complexity of biosilica deposition mechanisms. Factors such as morphotype-specific deposition processes, cell size, morphotype structure and composition, and the functional role of phytoliths in stress responses all may contribute to the observed variability. For instance, Bulliform cells, which are larger and more structurally involved in water stress mitigation, demonstrate greater sensitivity to changes in water availability. This sensitivity makes them more reliable indicators compared to Elongate and Stoma morphotypes. Nevertheless, the significant variability in δ^30^Si values across different plant tissues and morphotypes suggests that a broader genetic and environmental analysis is needed. Factors such as genetic diversity among sorghum varieties and micro-environmental conditions, like temperature and humidity during phytolith formation, could further elucidate the mechanisms behind Si uptake and distribution.

The study also offers further confirmation that sorghum actively transports silicic acid. Our data show that variations in Si absorption and deposition are influenced by environmental changes, specifically water stress. The non-uniform fractionation observed across different morphotypes suggests an active modulation of Si uptake and distribution, driven by physiological mechanisms rather than passive uptake alone. This active process appears to be modulated by environmental factors, such as water scarcity, which impact the relationship between water transpiration and Si transport. Such dynamics align with previous research and underscore the importance of considering water stress as a key driver of Si variability in plants.

Ultimately, this research provides a strong foundation for future studies to explore Si dynamics under environmental stress. The implications of our findings not only advance the field of plant physiology but also open new avenues for interdisciplinary research, linking agricultural science, ecology, and paleoclimate studies.

## Data Availability

The dataset generated during the current study are available in Zenodo repository at https://doi.org/10.5281/zenodo.14892282

## Abbreviations

fsLA-MC-ICP-MS: Femtosecond laser ablation multi-collector inductively coupled plasma-mass spectrometry
WW: Well-watered
WS: Water stressed
